# Epitope specificity of anti-Adrenomedullin antibodies determines efficacy of mortality reduction in a cecal ligation and puncture mouse model

**DOI:** 10.1186/2197-425X-1-3

**Published:** 2013-10-29

**Authors:** Joachim Struck, Frauke Hein, Siegmund Karasch, Andreas Bergmann

**Affiliations:** AdrenoMed AG, Neuendorfstr. 15a, Hennigsdorf, 16761 Germany; ICI GmbH, Berlin, 10999 Germany

**Keywords:** Adrenomedullin, Sepsis, Therapy, Animal model, Monoclonal antibodies

## Abstract

**Introduction:**

Adrenomedullin (ADM), a circulating vasodilatory peptide, plays an important role in the development of sepsis-associated hemodynamic and microcirculatory disorders. While administration of exogenous ADM had beneficial effects in several septic animal models, elevated ADM concentrations are associated with a bad outcome. This prompted us to test the effect of various anti-ADM antibodies in a cecal ligation and puncture (CLP) mouse model.

**Methods:**

To gain new potential compounds for the treatment or prevention of septic shock we followed an alternative strategy to influence the ADM system: High-affinity anti-ADM antibodies with different epitope specificities were developed and their antagonist activity *in vitro* and their ability to reduce mortality in a CLP mouse model were assessed.

**Results:**

An anti-ADM antibody directed against the N-terminus substantially increased the survival of mice in a CLP model (HR = 0.077 (CI = 0.0189 to 0.315), *p* = 0.0004), whereas other antibodies with similar affinities but different epitope specificities were much less potent. The efficacious antibody, in contrast to an anti-C-terminal antibody, only partially inhibited ADM agonist activity *in vitro*. Healthy mice were not negatively affected by the N-terminal antibody.

**Conclusions:**

An anti-N-terminal ADM antibody, as opposed to antibodies with other epitope specificities, strongly reduces mortality in CLP mice.

**Electronic supplementary material:**

The online version of this article (doi:10.1186/2197-425X-1-3) contains supplementary material, which is available to authorized users.

## Introduction

Despite advances in supportive care, each year 750,000 people develop sepsis and 225,000 die in the USA alone, and the incidence of sepsis is rising at rates between 1.5% and 8% per year [1–3]. Sepsis is a complex and dynamic condition initiated by an infectious stimulus and proceeding to an exaggerated systemic immune response [4, 5]. Several approaches in the past two decades to improve sepsis-associated mortality by novel therapies have had only limited only [6–10].

The peptide adrenomedullin (ADM) has been implicated in the development of septic shock: It is important in initiating the hyperdynamic response during the early stage of sepsis, and the reduced vascular responsiveness to ADM is associated with the transition from the hyperdynamic phase to the hypodynamic phase during the progression of sepsis [11–14].

ADM comprises 52 amino acids and is secreted into the blood stream from a variety of tissues [15–17]. ADM has multiple functions, the most prominent being its vasodilatory activity. It acts on vascular smooth muscle cells and vascular endothelial cells by binding to a G protein-coupled receptor system composed of the calcitonin receptor-like receptor (CRLR) and an accessory protein (receptor activity-modifying protein (RAMP) 2 or 3) via downstream cyclic adenosine monophosphate (cAMP) and NO signaling. In several preclinical studies exogenous ADM reduced mortality from septic shock and multiple organ failure (MOF) by reducing vascular hyperpermeability, preventing endothelial cell dysfunction and down-regulating the inflammatory response [12, 18–21].

Apparently, in contrast to the reported beneficial effects of ADM in sepsis, ADM has been categorized as a cardiac depressant factor in sepsis: ADM (22-52), an ADM receptor antagonist, improved the contractility of myocytes isolated from lipopolysaccharide (LPS)-treated rats. In addition, an anti-ADM antiserum improved the survival of LPS-treated rats [22].

Plasma levels of circulating ADM are relatively stable throughout various clinical conditions [23]. The strongest elevations have been observed in severe sepsis and septic shock, where increased ADM levels are associated with fatal outcome [24–26], suggesting that excessively high concentrations of ADM can be detrimental. In the present study, we assessed whether certain antibodies against ADM are potentially suitable therapeutic compounds to reduce sepsis-associated mortality in a cecal ligation and puncture mouse model, and how their efficacy in the animal model is associated with their affinities, epitope specificities, and antagonist activities.

## Material and methods

### Development of mouse monoclonal anti-ADM antibodies

Antipeptide antibodies were generated and selected by standard procedures (Unicus OHG, Karlsburg, Germany): Peptides were synthesized, as listed in Table 1 (JPT Technologies GmbH, Berlin, Germany). An additional N-terminal cysteine residue was added if no cysteine was present within the selected ADM-derived sequence. The peptides were conjugated to maleimide-activated bovine serum albumin (BSA). BALB/c mice were immunized with 100 μg of peptide-BSA conjugate per animal at days 0 and 14 (emulsified in 100 μL of complete Freund's adjuvant) and 50 μg at days 21 and 28 (in 100 μL of incomplete Freund's adjuvant). Three days before the fusion, each animal received 50 μg of the conjugate dissolved in 100 μL of saline, given as one intraperitoneal and one intravenous injection. Splenocytes from the immunized mice and cells of the myeloma cell line SP2/0 were fused with 1 mL of 50% polyethylene glycol. After washing, the cells were seeded in 96-well cell culture plates. Hybridomas were selected by growing in hypoxanthine-aminopterin-thymidine (HAT) medium (RPMI-1640 culture medium (Sigma-Aldrich, St. Louis, MO, USA) supplemented with 20% fetal calf serum and HAT Supplement (Life Technologies, Grand Island, NY, USA)). After 2 weeks, the HAT medium was replaced with HT medium for three passages followed by returning to the normal cell culture medium. The cell culture supernatants were primarily screened for antigen-specific IgG antibodies 3 weeks after fusion. The positive tested microcultures were transferred into 24-well plates for propagation. After retesting, the selected cultures were cloned and recloned using the limiting dilution technique and the isotypes were determined. Monoclonal antibodies were produced and purified by standard methods employing Protein A chromatography. The antibody purities were >95% based on SDS gel electrophoresis analysis. Fab and F(ab)_2_ fragments were generated by standard procedures. For antibody nomenclature, see Table 1.

**Table 1:** **Overview on monoclonal anti-ADM antibodies and their characteristics**

Antibody #	Immunogen	Amino acid position of immunogen in adrenomedullin	IgG subtype	Kd (M)	Maximal inhibition in bioassay (%)
NT-M	YRQSMNQGSRSNGCRFGTC	1-19	2a	5.8 × 10^-9^	24.0
MR-M	CTFQKLAHQIYQ	19-31	1	5.9 × 10^-9^	61.7
CT-M	CAPRNKISPQGY-NH_2_	Cys-40-50	2b	9.0 × 10^-9^	102.1
NT-H	YRQSMNNFQGLRSFGCRFGTC	1-21	1	1.6 × 10^-8^	25.5
MR-H	CTVQKLAHQIYQ	21-32	1	2.0 × 10^-9^	74.4
CT-H	CAPRSKISPQGY-NH_2_	Cys-42-52	1	1.1 × 10^-9^	100.7

The antibodies were named according to their epitope specificities as follows: NT, anti-N-terminal region; MR, anti-midregion; CT, anti-C-terminal region; and the suffixes M and H designate mouse and human ADM, respectively.

### Affinity constants

To determine the affinity of the antibodies to human and mouse ADM, respectively, the association and dissociation kinetics of ADM binding to immobilized antibody was determined by means of label-free surface plasmon resonance using a Biacore 2000 system (GE Healthcare Europe GmbH, Freiburg, Germany). Reversible immobilization of the antibodies was performed using an anti-mouse Fc antibody covalently coupled in high density to a CM5 sensor surface according to the manufacturer's instructions (mouse antibody capture kit; GE Healthcare). Antibody binding kinetics were determined by standard procedures, and affinity constants were calculated (Biaffin GmbH, Kassel, Germany).

### ADM bioassay

CHO-K1 cells expressing recombinant ADM receptor (CRLR + RAMP3 (FAST-027C; Euroscreen S.A., Brussels, Belgium) grown in media without antibiotic prior to the test were detached by gentle flushing with PBS-EDTA (5 mM EDTA), recovered by centrifugation, and resuspended in assay buffer (5 mM KCl, 1.25 mM MgSO_4_, 124 mM NaCl, 25 mM HEPES, 13.3 mM glucose, 1.25 mM KH_2_PO_4_, 1.45 mM CaCl_2_, 0.5 g/L BSA). Dose-response curves were obtained in parallel with the reference agonists human ADM and mouse ADM. After addition of the lysis buffer, cAMP was measured with an HTRF kit according to the instructions of the manufacturer (Cis-Bio International, Bagnols, France). The dose-dependent antagonist activity of anti-ADM antibodies was determined in the presence of human ADM or mouse ADM, respectively, either one at a predetermined concentration yielding 80% of the maximal stimulation obtainable for the cAMP response: 6 μL of the reference agonist (human (5.63 nM) or mouse (0.67 nM) ADM) was mixed with 6 μL of the test samples at different antibody dilutions of 100, 20, 4, 0.8, and 0.16 μg/mL. After incubation for 60 min at room temperature, 12 μL of cells (2,500 cells/well) were added. The plates were incubated for 30 min at room temperature, and cAMP was measured as described above. Antagonist activity of anti-ADM antibodies was calculated as percentage of inhibition relative to the maximal inhibition obtainable with the reference antagonist peptide ADM (22-52).

### Sepsis model (cecal ligation puncture)

Twelve- to fifteen-week-old male C57Bl/6 mice (Charles River Laboratories, Cologne, Germany) were used for the study (Phenos GmbH, Hannover, Germany). Peritonitis had been surgically induced under light isoflurane anesthesia. Incisions were made into the left upper quadrant of the peritoneal cavity (normal location of the cecum). The cecum was exposed and a tight ligature was placed around the cecum with sutures distal to the insertion of the small bowel. One puncture wound was made with a 24-gauge needle into the cecum, and small amounts of cecal contents were expressed through the wound. The cecum was replaced into the peritoneal cavity and the laparotomy site was closed. Finally, the animals were returned to their cages with free access to food and water. Five hundred microliters of saline was given s.c. as fluid replacement.

### Efficacy test

Using this CLP model, three anti-mouse ADM antibodies (NT-M, MR-M, and CT-M) were tested for their ability to modify the 14-day mortality rate in comparison to vehicle (PBS buffer) and control compound (unspecific IgG). Ten animals per compound were used. Compounds were applied i.v. immediately prior to surgery (10 μL/g body weight, 0.2 mg/mL). Mice were monitored twice daily. In a follow-up experiment, Fab- and F(ab)_2_ fragments of NT-M were tested analogously.

### Safety test

Twelve- to fifteen-week-old male C57Bl/6 mice (Charles River Laboratories, Germany) were used for the study. Six mice were treated with NT-M anti-ADM antibody (10 μL/g body weight; 3.0 mg/mL). As control, six mice were treated with PBS (10 μL/g body weight). Survival and physical condition were monitored for 14 days.

### Ethics

For all animal experiments described in this manuscript, approval was obtained according to the requirements of the respective German law (Tierschutzgesetz (TierSchG)) from the Niedersächsisches Landesamt für Verbraucherschutz und Lebensmittelsicherheit (LAVES). Our study has been purely experimental and did not involve any human samples.

### Statistics

Statistical significance was calculated by Student's *t* test. For survival analysis, Kaplan-Meier curves were generated and log-rank test for significance was performed. GraphPad Prism 4 software (GraphPad Software, Inc., La Jolla, CA, USA) was used.

## Results

### Anti-ADM antibodies

**Figure 1:**
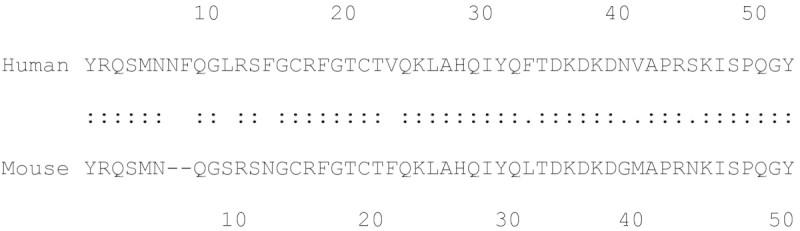
**Amino acid sequences of human and mouse Adrenomedullin.** Monoclonal antibodies were developed against peptides representing positions 1–21, 21–32 and 42–52 of human Adrenomedullin and against peptides representing positions 1–19, 19–31, 40–50 of mouse Adrenomedullin.

Several mouse monoclonal antibodies against the N-terminal, midregional, and C-terminal moieties of mouse ADM (NT-M, MR-M, CT-M) and human ADM (NT-H, MR-H, CT-H) were developed (Figure 1, Table 1). The affinity constants of the antibodies were in the range of 1.1 × 10^-9^ to 1.6 × 10^-8^ M (Table 1).

**Figure 2:**
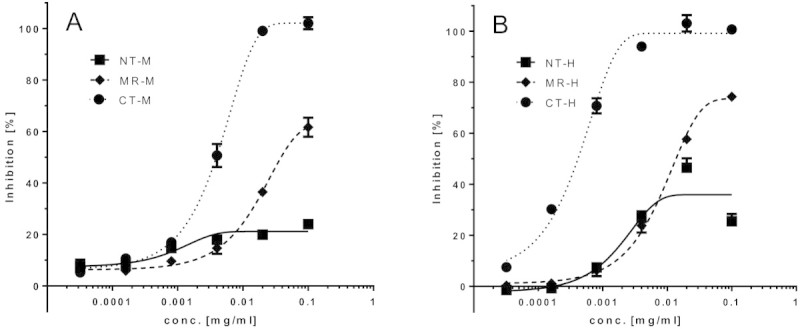
**Dose/response curves for various anti-ADM antibodies affecting ADM-induced cAMP response in an ADM bioassay (antagonist activity).** The monoclonal antibodies used were directed against the N-terminus (NT-M), mid-region (MR-M) and C-terminus (CT-M) of mouse ADM in the presence of 0.67 nM mouse ADM **(panel A)**, and directed against the N-terminus (NT-H), mid-region (MR-H) and C-terminus (CT-H) of human ADM in the presence of 5.63 nM human ADM **(panel B)**.

The agonist and antagonist activities of the antibodies were tested in an established ADM bioassay system employing a CHO cell line overexpressing the human recombinant ADM receptor (CRLR + RAMP3) with a cAMP readout. None of the antibodies exhibited agonist activity in the bioassay (data not shown). The antibodies showed different dose-dependent antagonist activity profiles (Figure 2, Table 1). Surprisingly, the observed differences were dependent on the epitope specificity rather than on the affinity of the antibodies, for both the anti-human ADM and the anti-mouse ADM antibodies: The maximal obtainable inhibition of the ADM-induced cAMP response was 100% for the antibodies directed against the C-terminal moiety of ADM, around 70% for the antibodies directed against the midregional moiety of ADM, and around 25% for the antibodies directed against the N-terminal moiety of ADM (Figure 2, Table 1).

**Figure 3:**
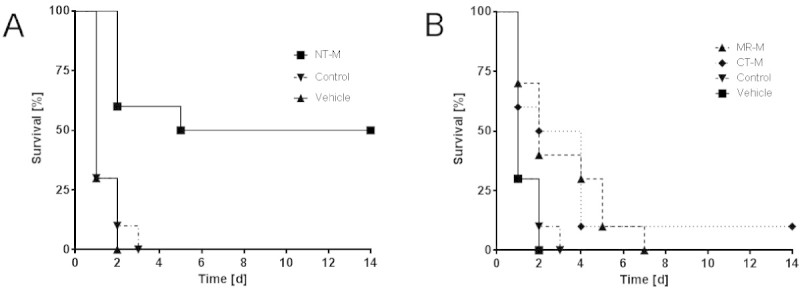
**Survival rates of CLP mice treated with various antibodies.** The monoclonal antibodies used were directed against the N-terminus (NT-M) **(Panel A)**, and mid-region (MR-M) and C-terminus (CT-M) of mouse ADM **(Panel B)**. An unspecific mouse IgG was used as control (shown in Panels **A and B**). For statistics see main text.

**Figure 4:**
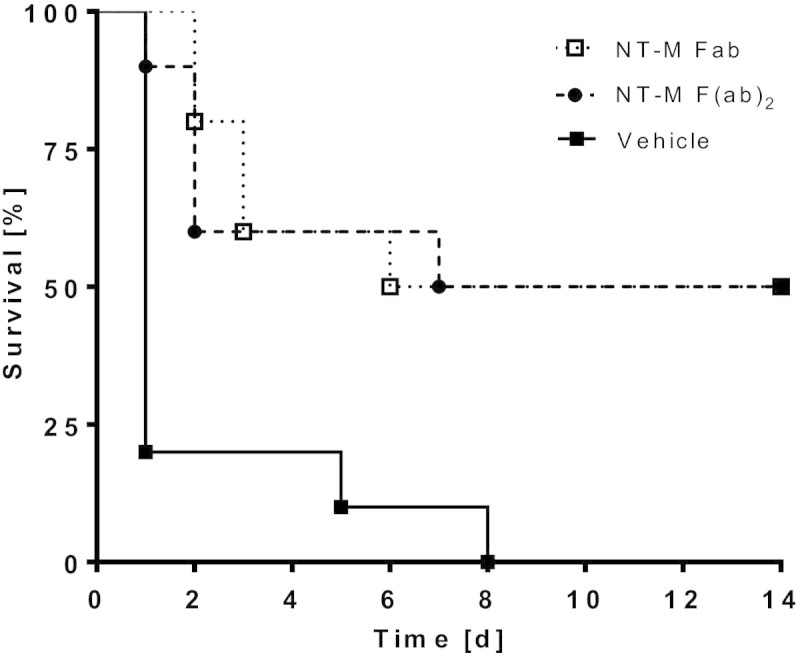
**Survival rates of CLP mice treated with NT-M antibody fragments.** Fab and F(ab)_2_ fragments of the antibody directed against the N-terminus (NT-M) of ADM were tested. For statistics see main text.

The anti-mouse ADM antibodies were tested in a CLP sepsis mouse model for their ability to reduce mortality. The observation period was 14 days. The doses of antibodies were chosen so that concentrations *in vivo* should by far exceed on a molar basis the expected concentrations of endogenous plasma ADM. In the control groups (vehicle buffer or unspecific control antibody), most of the animals had died already on day 1 (Figure 3). The antibodies against the midregion and C-terminus of ADM improved survival slightly, but significantly, when compared to either vehicle or control (MR-M vs. vehicle: HR = 0.182 (CI = 0.760 to 0.043), *p* = 0.0195; MR-M vs. control: HR = 0.201 (CI = 0.051 to 0.789), *p* = 0.0215; CT-M vs. vehicle: HR = 0.182 (CI = 0.766 to 0.043), *p* = 0.0202; CT-M vs. control: HR = 0.1796 (CI = 0.044-0.733), *p* = 0.0167). A strong and sustained improvement of survival was obtained with the antibody against the N-terminus of ADM: 50% of the animals treated with this antibody survived the CLP procedure over the observation period of 14 days (Figure 3) (NT-M vs. vehicle: HR = 0.068 (CI = 0.291 to 0.0159), *p* = 0.0003; NT-M vs. control: HR = 0.07717 (CI = 0.0189 to 0.315), *p* = 0.0004). The improvement of survival for the antibody against the N-terminus of ADM was significantly higher than for the anti-midregion and the anti-C-terminal antibody (NT-M vs. MR-M: HR = 0.212 (CI = 0.712 to 0.063), *p* = 0.0122; NT-M vs. CT-M: HR = 0.285 (CI = 0.0823 to 0.979), *p* = 0.0462). Similar results were obtained in a follow-up experiment with Fab and F(ab)_2_ fragments of the antibody against the N-terminus of ADM (Figure 4; Fab: HR = 0.273 (CI = 0.043 to 0.356), *p* = 0.0017; F(ab)_2_: HR = 0.298 (CI = 0.044 to 0.354), *p* = 0.0025). When the antibody against the N-terminus of ADM was administered to healthy mice even at a 15-fold-higher concentration than that in the CLP experiment, all animals survived and the physical condition of the animals was not impaired compared to control animals (data not shown).

When the antibody against the N-terminus of ADM was administered to healthy mice even at a 15-fold higher concentration than in the CLP experiment, all animals survived and the physical condition of the animals was not impaired compared to control animals (data not shown).

## Discussion

In this study, we report that an antibody directed against the N-terminus of ADM substantially reduces mortality in a septic animal model, whereas antibodies against other regions of ADM are far less efficacious. Anti-N-terminal antibodies only partially inhibit ADM agonist function in a bioassay.

It has been known that plasma ADM levels strongly increase with the severity of sepsis, severe sepsis, and septic shock, and that elevated levels are highly prognostic for fatal outcome [24–26]. This had suggested that ADM plays a role in the cascade of events happening during the progression of sepsis, leaving open the question, though, of whether ADM represents the 'fire’ or the 'firemen’ , whether it is friend or foe in this process. ADM has been shown to be a key player in initiating the hyperdynamic response during the early stage of sepsis [12–14]. When sepsis proceeds to hypodynamic shock, vascular responsiveness to ADM fades, suggesting that reduced ADM function could be causal for progression of the disease. Consequently, supplementation of exogenous ADM has been tested in animal models as a possibility to reduce mortality from sepsis [12, 18–21]. These attempts were successful. However, concerns have been raised with respect to the safety of this approach as ADM, due to its strong vasodilatory activity, might be harmful in certain situations, and thus, the therapeutic regime might be difficult to control [27]. Additionally, ADM as a small peptide is prone to proteolytic degradation and - as plasma protease activities increase and vary in sepsis patients [28] - it might not be an ideal drug structure by concept. Moreover, at least the group of Wang is convinced that ADM alone is not sufficiently efficacious, but coadministration of complement factor H, an ADM binding protein, is necessary [12]. Development of such a two-compound drug appears challenging in several aspects.

ADM might not be beneficial under all circumstances in the progression of sepsis: *In vitro* experiments suggest that ADM has negative inotropic effects and cause impairment of cardiac function in sepsis: The contractility of cardiac myocytes isolated from LPS-treated rats was reduced compared to that in control animals, and addition of ADM (22-52), an ADM receptor antagonist peptide, reversed this effect [22]. With an anti-ADM antiserum, mortality of LPS-treated rats could be reduced [22]. It was interpreted that during the progression of sepsis, the role of ADM reverses from that of a compensatory mediator to a perpetuator of cardiodepression [29]. The anti-ADM antiserum used to successfully reduce mortality in septic rats was not further characterized; no information was gained concerning the concentration of anti-ADM antibodies in the antiserum, their affinities, their epitope specificities, and their agonist and antagonist activities.

We set out to systematically develop and characterize monoclonal antibodies directed against various epitopes of ADM and investigate their potential utility as drugs to reduce sepsis-associated mortality in a mouse CLP model. Antibodies against the C-terminal moiety of ADM were capable of fully inhibiting the ADM-induced cAMP response in an ADM receptor bioassay. This could have been expected as ADM (22-52) is known to be an effective ADM receptor antagonist. Less expected was our finding that antibodies against the N-terminal moiety of ADM inhibited ADM agonist activity maximally by only around 25%. The level of inhibition did not change over almost three orders of magnitude of antibody concentration tested. It was exactly such an anti-N-terminal antibody as well as Fab and F(ab)_2_ fragments thereof, which substantially reduced mortality in a mouse CLP model. In contrast, an anti-C-terminal antibody did not strongly improve survival in this animal model. Thus, promotion of survival apparently requires on one hand a certain minimum level of functional ADM and on the other hand the reduction of excessively high concentrations of ADM. It appears paradoxical that both supplementation with ADM, as shown previously [12, 18–21], and antibodies against ADM, as we show here, can reduce mortality in septic animal models. We can only speculate about the mechanistic background of these observations: As ADM has an *in vivo* half-life of only 22 min [30], a relatively short increase of ADM levels, especially in the early phase of sepsis progression, might be beneficial. The reduction of ADM functionality by an anti-N-terminal antibody might be more relevant in the later phase, when excessive endogenous ADM production is associated with fatal outcome. Our results indicate that complete inhibition of ADM agonist activity during sepsis is not very efficacious, if at all, to improve survival. The mechanism leading to a substantially improved survival rate of CLP mice when treated with an anti-N-terminal antibody is not completely clear. We hypothesize that binding of the anti-N-terminal antibody to ADM still allows receptor binding, but less efficiently, and thus reduces the functionality of ADM so that excess levels, which have been suggested to become harmful during the progression of sepsis, then get functionally neutralized to a certain limited extent. Partial functional inhibition of ADM, on the other hand, leaves sufficient ADM available, which is required in the early hyperdynamic phase of sepsis and possibly later as well. It is not excluded that besides the functional modulation of ADM, the anti-N-terminal antibody influences the proteolytic decay of circulating ADM, since ADM is N-terminally susceptible to proteolytic degradation [31], and thereby prolongs its half-life and bioavailablity. In any case, the use of a defined anti-N-terminal antibody constitutes a novel approach to modulate the ADM pathway during the progression of sepsis, which has some obvious advantages over previously applied concepts involving ADM as a drug component: An antibody is less prone to proteolytic degradation than ADM peptide; elevated and varying proteolytic activity might in an uncontrolled manner affect peptide integrity in the circulation of sepsis patients. ADM is a very sticky molecule [32], and this might add risk in the drug production and application processes. Thirdly, concerns have been raised against the use of ADM as a drug since it is known as a strong vasodilator, and thus it might be difficult to define the therapeutic window and avoid potentially harmful effects outside this window. In contrast, the anti-N-terminal antibody appears safe as it did not impair healthy mice, even at a 15-fold-higher concentration than that successfully used in the CLP model. Presumably, this is due to a relatively small modulation of the ADM system. Finally, the development of monoclonal antibodies as drugs is a standard process nowadays. If it turns out that, as Wang's group suggests, in fact complement factor H must be coadministered with ADM to gain sufficient efficacy, then this represents another challenge for drug development, which we do not face with the development of a therapeutic antibody.

## Conclusions

In conclusion, we have shown in an established septic mouse model a strongly beneficial effect on survival by use of an anti-N-terminal ADM antibody. The effect is due to its epitope specificity and is associated with partial inhibition of ADM agonist function. The results of this pilot study are a basis to further develop an alternative, advantageous way for drug development modulating the ADM pathway to finally reduce mortality during the progression of sepsis, which continues to be a strong unmet clinical need.

## Key messages

The following are the key messages derived from this study:

-  Anti-ADM antibodies have different antagonist activity *in vitro* depending on their epitope specificity.
-  Antagonist activity is strong for anti-C-terminal antibodies, medium for anti-midregional antibodies and weak for anti-N-terminal antibodies.
-  Anti-ADM antibodies are significantly but differently efficacious in reducing mortality in CLP-treated mice depending on their epitope specificity.
-  The anti-N-terminal antibody reduces mortality substantially, whereas antibodies against other regions of ADM are less potent.

## Authors’ original submitted files for images

Below are the links to the authors’ original submitted files for images. Authors’ original file for figure 1 Authors’ original file for figure 2 Authors’ original file for figure 3 Authors’ original file for figure 4

## Abbreviations

AMBP-1: Adrenomedullin binding protein 1
ADM: Adrenomedullin
BSA: Bovine serum albumin
CT-H: Mouse monoclonal antibody against the carboxyterminal region of human adrenomedullin
CT-M: Mouse monoclonal antibody against the carboxyterminal region of mouse adrenomedullin
CLP: Cecal ligation and puncture
CRLR: Calcitonin receptor-like receptor
LPS: Lipopolysaccharide
MOF: Multiple organ failure
MR-H: Mouse monoclonal antibody against the midregion of human adrenomedullin
MR-M: Mouse monoclonal antibody against the midregion of mouse adrenomedullin
NT-H: Mouse monoclonal antibody against the aminoterminal region of human adrenomedullin
NT-M: Mouse monoclonal antibody against the aminoterminal region of mouse adrenomedullin
PBS: Phosphate buffered saline
RAMP: Receptor-activity modifying protein.
