# Supplementary material for: Epitope specificity of anti-Adrenomedullin antibodies determines efficacy of mortality reduction in a cecal ligation and puncture mouse model
Source: Intensive Care Med Exp. 2013 Oct 29;1:3. doi: 10.1186/2197-425X-1-3 (PMC4796695; doi:10.1186/2197-425X-1-3)
Supplement: Supplementary file 1 — Authors’ original file for figure 1 [file 40635_2013_22_MOESM1_ESM.pdf]

```
              10        20        30        40        50
Human YRQSMNNFQGLRSFGCRFGTCTVQKLAHQIYQFTDKDKDNVAPRSKISPQGY
      ::::::  :: :: :::::::: :::::::::.::::::..:::.:::::::
Mouse YRQSMN--QGSRSNGCRFGTCTFQKLAHQIYQLTDKDKDGMAPRNKISPQGY
                10        20        30       40         50
```
