# Supplementary figures and images for: Epitope specificity of anti-Adrenomedullin antibodies determines efficacy of mortality reduction in a cecal ligation and puncture mouse model
Source: Intensive Care Med Exp. 2013 Oct 29;1:3. doi: 10.1186/2197-425X-1-3 (PMC4796695; doi:10.1186/2197-425X-1-3)

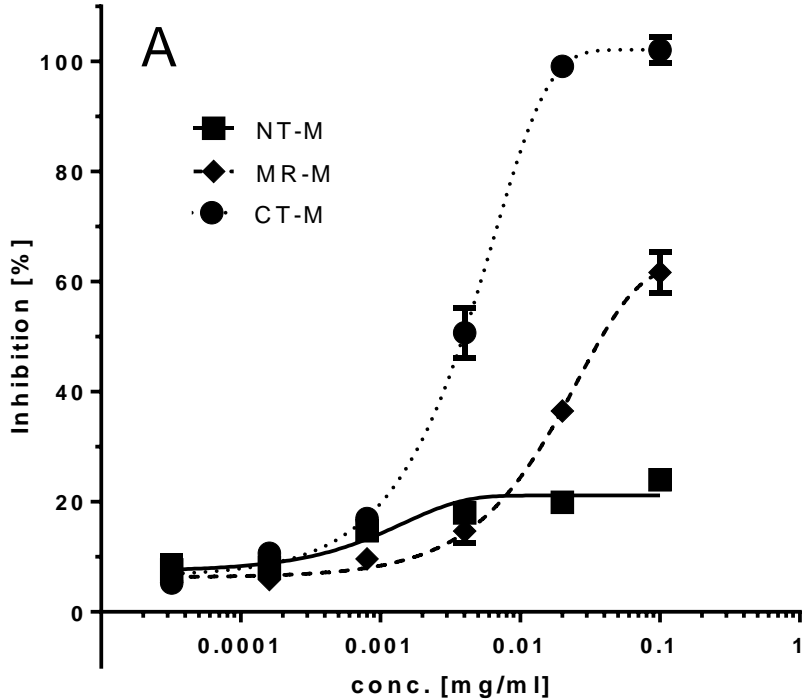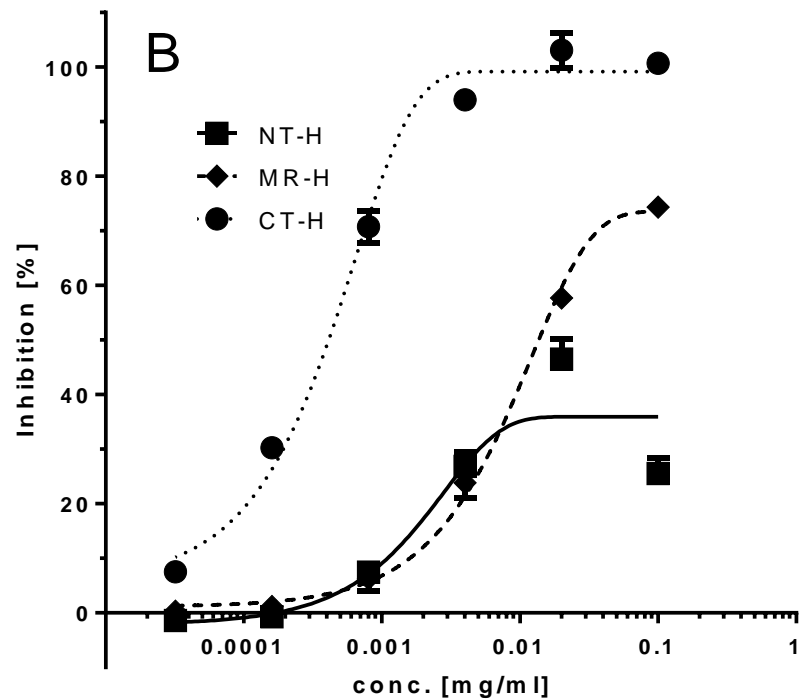

Supplement: Supplementary file 2 — Authors’ original file for figure 2 [file 40635_2013_22_MOESM2_ESM.pdf]

**A**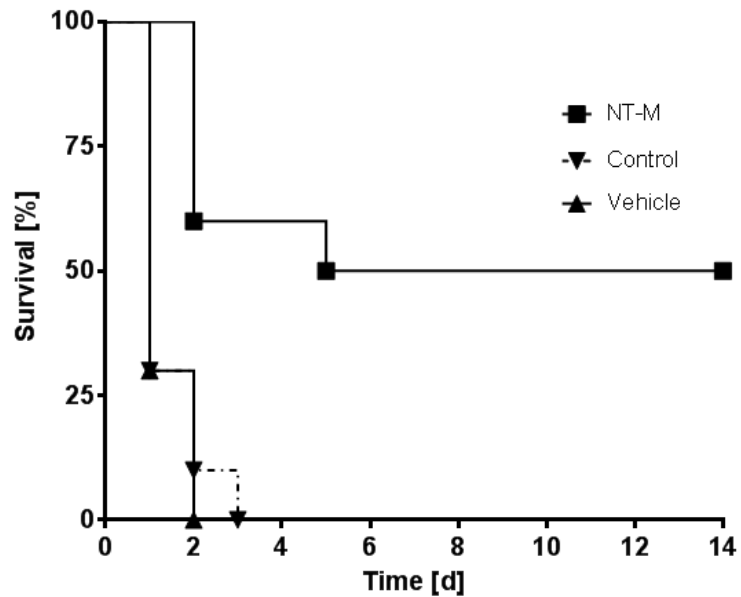**B**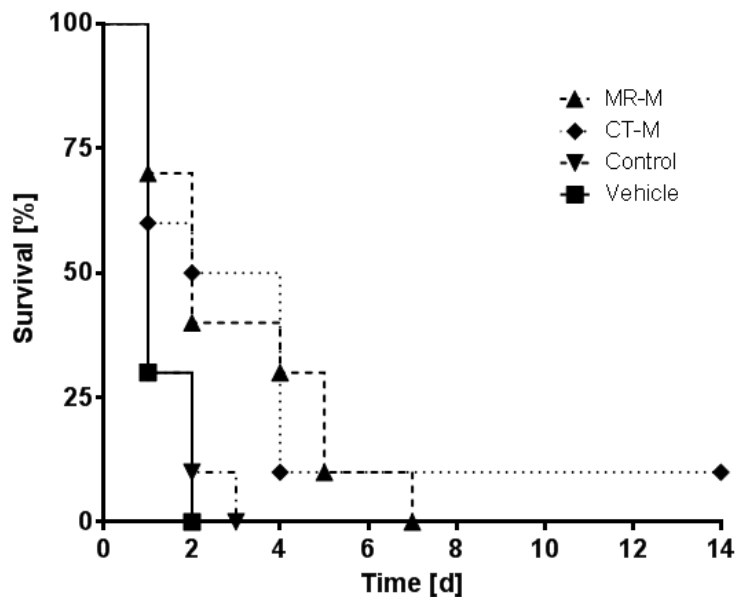

Supplement: Supplementary file 3 — Authors’ original file for figure 3 [file 40635_2013_22_MOESM3_ESM.pdf]

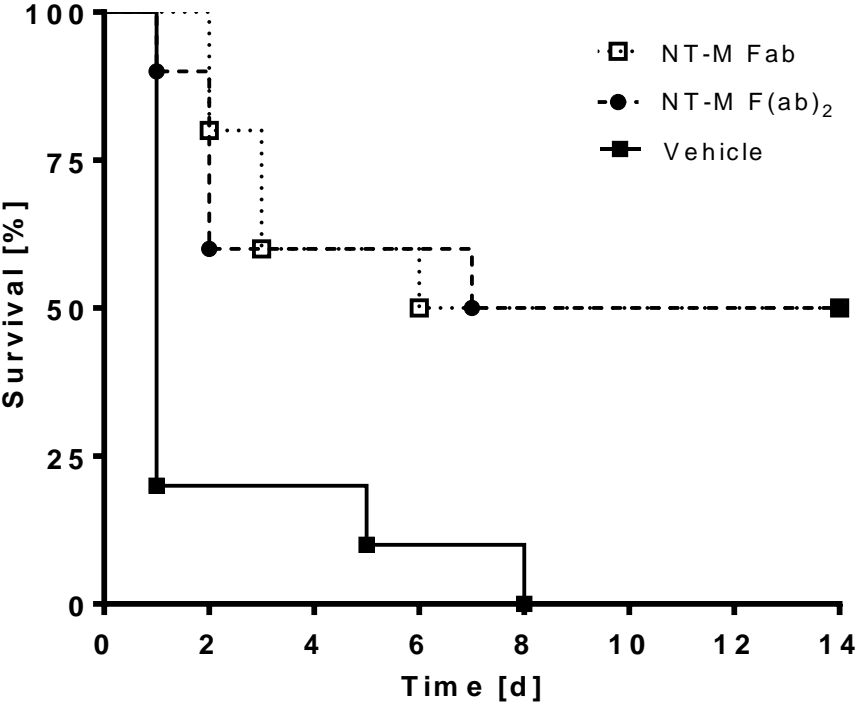

Supplement: Supplementary file 4 — Authors’ original file for figure 4 [file 40635_2013_22_MOESM4_ESM.pdf]
